# Halogenated Analogs to Natural A-Type Proanthocyanidins: Evaluation of Their Antioxidant and Antimicrobial Properties and Possible Application in Food Industries

**DOI:** 10.3390/molecules29153622

**Published:** 2024-07-31

**Authors:** Antonio Cobo, Alfonso Alejo-Armijo, Daniel Cruz, Joaquín Altarejos, Sofía Salido, Elena Ortega-Morente

**Affiliations:** 1 Department of Health Sciences, Faculty of Experimental Sciences, University of Jaén, Campus of International Excellence in Agri-Food (ceiA3), 23071 Jaén, Spain; acmolinos@ugr.es (A.C.); dcsaez@ujaen.es (D.C.); 2 Department of Inorganic and Organic Chemistry, Faculty of Experimental Sciences, University of Jaén, Campus of International Excellence in Agri-Food (ceiA3), 23071 Jaén, Spain; aalejo@ujaen.es (A.A.-A.); jaltare@ujaen.es (J.A.)

**Keywords:** A-type proanthocyanidin analogs, flavylium chemistry, antimicrobial activity, antibiofilm activity, antioxidant activity

## Abstract

A description of new antimicrobial agents suitable for food industries has become necessary, and natural compounds are being considered as promising sources of new active derivatives to be used with the aim of improving food safety. We have previously described desirable antimicrobial and antibiofilm activities against foodborne bacteria by analogs to A-type proanthocyanidins (PACs) with a nitro (NO_2_) group at carbon 6 of the A-ring. We report herein the synthesis of eight additional analogs with chloro and bromo atoms at the A-ring and the systematic study of their antimicrobial and antioxidant activities in order to evaluate their possible application as biocides or food preservatives, as well as to elucidate new structure–activity relationships. The results from this study show that halogenated analogs to natural A-type proanthocyanidins rise above the nitro derivatives previously reported in their antimicrobial activities. Gram-positive bacteria are the most sensitive to all the analogs and combinations assayed, showing MICs from 10 to 50 μg/mL in most cases, as well as reductions in biofilm formation and the disruption of preformed biofilms of at least 75%. Some structure–activity relationships previously described have also been corroborated. Analogs with just one OH group at the B-ring show better antimicrobial activities than those with two OH groups, and those analogs with two or three OH groups in the whole structure are more active than those with four OH groups. In addition, the analogs with two OH groups at the B-ring and chloro at the A-ring are the most effective when antibiofilm activities are studied, especially at low concentrations.

## 1. Introduction

The frequent episodes of foodborne diseases have become a major public health concern, associated with high morbidity and mortality rates [1]. A report published by the Centers for Disease Control and Prevention (CDC) of the United States (US) highlighted this issue, pointing out foodborne bacteria, mainly *Escherichia* and *Listeria*, as responsible for the high rates of outbreaks, illnesses, hospitalizations, and deaths reported during 2013–2017 [2]. Moreover, around 80% of microbial infections were related to the presence of microbial biofilms on surfaces of materials [3]. High rates of resistance detected in foodborne bacteria render the description of new antimicrobial agents necessary, and natural compounds are being considered as promising sources of new antimicrobial derivatives to be used in food industries with the aim of developing antibiofilm materials for improved food safety [4]. Previous research on food packaging using A-type proanthocyanidins (PACs) has revealed the effect of these compounds on the physical, antioxidant and antimicrobial properties of chitosan-based films, mainly improving different physical properties such as the oxygen barrier ability, UV–vis light barrier ability, tensile strength and thermal stability of chitosan-based films [5].

We have also previously described desirable antimicrobial and antibiofilm activities against foodborne bacteria by analogs to A-type proanthocyanidins with electron-donating and electron-withdrawing groups at the A-ring [6] and with a nitro (NO_2_) group at carbon 6 of the A-ring [7]. Both investigations allowed us to conclude that electron-withdrawing groups at the A-ring (NO_2_) enhanced the antimicrobial properties of the analogs evaluated [7]. Moreover, some structure–activity relationships were derived from this study with NO_2_ derivatives [7]: (a) one OH group at the B-ring improved the antimicrobial activity compared to two OHs at the same ring; (b) two or three OH groups in the whole structure improved the antimicrobial activity compared to four OHs in total; (c) two OH groups at the B-ring, two OH groups at the D-ring and a methyl group at the C-ring were the most effective combination, especially at low concentrations, to enhance the inhibition of biofilm formation and the disruption of preformed biofilms.

**Figure 1:**
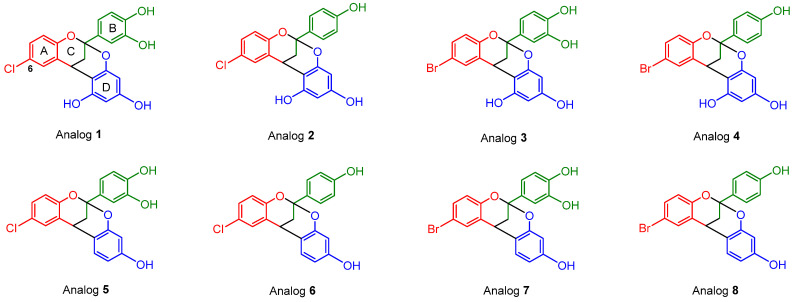
Analogs to A-type PACs synthesized and evaluated in this study.

**Scheme 1:**
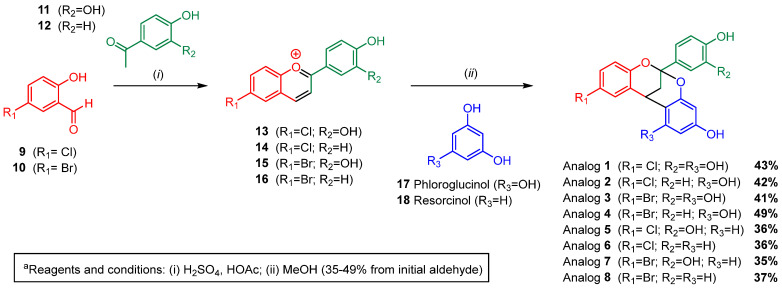
Synthetic route to prepare A-type proanthocyanidin analogs **1**–**8** from flavylium salts **13**–**16** and phloroglucinol (**17**)/resorcinol (**18**) ^a^.

We report herein the synthesis of eight additional analogs with chloro and bromo atoms at the A-ring and the systematic study of their antimicrobial and antioxidant activities in order to evaluate their possible application as biocides or food preservatives, as well as to elucidate new structure–activity relationships that may explain their activities and broaden the field of study on these type of compounds. These analogs also contain an electron-withdrawing group at carbon 6 of the A-ring (similar to nitro derivatives reported before [7]), which could guarantee the activity of these new derivatives (Figure 1). They were synthesized by the nucleophilic attack of phloroglucinol or resorcinol on flavylium salts, which were prepared through the acid-catalyzed condensation of the corresponding 4-halogen-salicylaldehyde with acetophenone derivatives (Scheme 1).

## 2. Results and Discussion

### 2.1. Synthesis of Analogs ***1***–***8***

Scheme 1 shows the synthetic methodology used for preparing analogs **1**–**8**. These compounds have been synthetized in a two-step process previously used and optimized by us [8]. Briefly, the synthesis began with the acid-catalyzed condensation of the salicylic aldehyde derivatives (**9**–**10**) with acetophenone derivatives (**11**–**12**), which yielded flavylium salts **13**–**16**. In the second step, these species were allowed to react with phloroglucinol (**17**) or resorcinol (**18**) in absolute methanol at 50 °C to yield analogs **1**–**8** in moderate yields (35–49% from the initial aldehyde).

As expected, the major nucleophilic character of phloroglucinol (**17**) with respect to resorcinol (**18**) was reflected in the yields obtained. This behavior was previously observed by us [8]. In this sense, analogs with resorcinol moiety were formed with slightly lower yields (analogs **5**–**8**; R = 35–37%) than the ones obtained when phloroglucinol scaffold was used as π-nucleophile (analogs **1**–**4**; R = 41–49%). The ^1^H NMR and ^13^C NMR data of analogs **1**–**5** and **7** agreed with those reported in the literature [8,9]. Analogs **6** and **8** were new compounds and are reported here for the first time.

### 2.2. Antioxidant Activity

The antioxidant activity of analogs **1**–**8** was evaluated by two different methods: (a) a DPPH radical-scavenging assay, and (b) a Rancimat assay (Table 1). Regarding the first method, analogs **1**, **3**, **5** and **7**, with a catechol moiety at the B-ring, showed a high antioxidant ability similar to that of Trolox, a known reference antioxidant frequently used [10]. On the other hand, analogs **2**, **4**, **6** and **8**, with only one OH group at the B-ring, did not show such scavenging behavior, with EC_50_ values between 15 and 40 times higher than those of analogs with two vicinal OH groups (**1**, **3**, **5**, **7**) (Table 1). Regarding the Rancimat method, a parallel behavior has been observed with respect to the DPPH assay, since analogs **1**, **3**, **5** and **7** showed protection factors (PF) higher than those of analogs **2**, **4**, **6** and **8** (Table 1). This means that the presence of the catechol sub-structure in compounds **1**, **3**, **5** and **7** gave oxidative stability to the vegetal oil used in the assay (refined soybean oil), whereas compounds **2**, **4**, **6** and **8**, with PF values close to 1, hardly protected that oil from oxidation. The reference antioxidant used in the Rancimat test was Cinnamtannin B-1 (C-B1), the natural antioxidant that inspired the synthesis of 2,8-dioxabicyclo[3.3.1]nonane derivatives like **1**–**8** and others previously reported by us [7]. The PF value of C-B1 was intermediate between one group of analogs and another. At the second level, it also seemed that the presence of the phloroglucinol moiety in the structure was related with a slight enhancement in the antioxidant capacity, at least in the DPPH assay. This behavior can be observed in the EC_50_ column (Table 1) for analogs with only one OH group at the B-ring (**2**, **4**, **6**, **8**), for which the antioxidant activity of analogs with phloroglucinol (**2**, **4**) was at least two–three times higher than that of compounds with resorcinol (**6**, **8**).

### 2.3. Antimicrobial Activity

Table 2a shows growth inhibition diameters of the compounds on especially resistant strains from organic foods. Analogs **4**, **8** and **6** showed the best results, inducing analog **4** at 1000 μg/mL growth inhibition diameters of 40 mm and 30 mm on strains UJA29o and UJA7m, both of them identified as *P. agglomerans*. This compound also showed zones of inhibition of 20 mm on strains *E. faecium* UJA11c, *K. terrigena* UJA32j, *S. aureus* UJA34f and *L. casei* UJA35h. Analog **8** showed growth inhibition diameters of 20 mm against *E. faecium* UJA11c, *P. agglomerans* UJA29o, *K. terrigena* UJA32j, *S. aureus* UJA34f and *Salmonella* sp. UJA40l and of 15 mm against *S. saprophyticus* UJA27g and *L. casei* UJA35h. Moreover, 20 mm diameter growth inhibition was also detected when analog **6** was added to plates seeded with *S. aureus* UJA34f, *L. casei* UJA35h and *Salmonella* sp. UJA40k.

When the synthesized analogs were faced with strains from type-culture collections, the best effects were found on the strain *S. aureus* CECT 976, with diameters of inhibition zones of 30 mm for analogs **2** and **6** at 100 μg/mL and analogs **4** and **8** at 10 μg/mL, followed by the strain *S. aureus* CECT 828, which showed diameters of inhibition zones of 80 mm, 70 mm and 30 mm when faced with 100 μg/mL of analogs **4**, **8** and **2**, respectively. Zones of inhibition of 12 mm and 11 mm on the strains *S. aureus* CECT 828 and *S. aureus* CECT 976, respectively, were also found after incubation with analog **1**. Positive results were also shown for *Listeria innocua* CECT 910, with 30 mm of zone of inhibition for analog **2** at 100 μg/mL as well as for analogs **4** and **8** at 10 μg/mL. Analogs **4** and **8** also showed zones of inhibition of 10 to 20 mm on Gram-negative strains from type-culture collections (*E. coli* and *Salmonella*) at 100 μg/mL. 

**Table 2:** Growth inhibition diameters (mm) of analogs **1**–**8** against target strains.

						**(a)**							
**Analog**	**μ** **g/mL**	**UJA7m**	**UJA11c**	**UJA11e**	**UJA27g**	**UJA27q**	**UJA29o**	**UJA32j**	**UJA34f**	**UJA35h**	**UJA37p**	**UJA40k**	**UJA40l**
**1**	1000					<10			14	10			
	100					<10			<10				
**2**	100		10	10	10	10			20			15	
	10								10	10			
**3**	1				10	14			18	12			
	100					<10			12				
**4**	1000	30	20	10	10	10	40	20	20	20	10	10	10
**5**	1000					10			12	10			
**6**	100		10	10	10	10			20	20		20	
**7**	1000				11	13			19	12			
	100								10				
**8**	100	10	20	10	15	10	20	20	20	15	10	10	20
						**(b)**							
**Analog**	**μ** **g/mL**	***S. aureus*** **CECT 828**	***S. aureus*** ***CECT 976***		***L. innocua*** **CECT 910**	***E. coli*** **CCUG 47553**	***E. coli*** **CCUG 47557**		***S. enterica*** **CECT 4300**	***S. enterica*** **CECT409**	***S. enterica*** **CECT 4395**		***S. enterica*** **CECT 915**
**1**	100	12	11										
**2**	100	30	30		30	10							
	10	10											
**3**	100		<10										
**4**	100	80	60		60	20	15		10	20	20		10
	10	30	30		30								
**5**	100	<10	<10		<10								
**6**	100	10	30		10								
**7**	100	<10	<10										
**8**	100	70	40		70	20	20		10	10	10		10
	10		30		30								

(a) Resistant strains from organic foods; (b) strains from type-culture collections.

Among all the strains analyzed, those with the highest sensitivity to all the analogs were *S. aureus* CECT 828, *S. aureus* CECT 976, *S. aureus* UJA34f and *B. cereus* UJA27q, which showed zones of inhibition when faced with all (or all but one) of the compounds.

Minimal inhibitory concentration (MIC) values obtained for each analog (Table 3a,b) corroborated the preliminary data. Among the strains from organic foods, *S. saprophyticus* UJA27g, *B. cereus* UJA27q, *S. aureus* UJA34f and *L. casei* UJA35h were the most sensitive to all the analogs, showing MICs from 10 to 50 μg/mL in all cases. MICs ranging from 10 to 50 μg/mL were also found for analogs **2**, **4**, **6** and **8** against *P. agglomerans* UJA7m, *E. faecium* UJA11c, *E. casseliflavus* UJA11e, *Salmonella* sp. UJA40l and *Salmonella* sp. UJA40l. Analogs **4** and **8** also showed MICs of 50 μg/mL against *P. agglomerans* UJA29o and *K. terrigena* UJA32j.

Among strains from type-culture collections, MICs of 10 μg/mL were found for analogs **2**, **4**, **6** and **8** against *S. aureus* CECT 828 and *S. aureus* CECT 976, and for analogs **4**, **6** and **8** against *L. innocua* CECT 910. MICs of 50 μg/mL were obtained for analogs **1**, **3**, **5** and **7** against *S. aureus* CECT 828 and *S. aureus* CECT 976, and for analogs **2**, **5** and **7** against *L. innocua* CECT 910. An amount of 50 μg/mL was also the MIC value found for analog **2** against *E. coli* CCUG 47553 and for analogs **2** and **4** against *S. enterica* CECT 915. The MICs for all other Gram-negative-type strains analyzed were above 1000 μg/mL for all of the analogs tested.

**Table 3:** MICs of analogs **1**–**8** against target strains (μg/mL).

						**(a)**						
**Analog**	**UJA7m**	**UJA11c**	**UJA11e**	**UJA27g**	**UJA27q**	**UJA29o**	**UJA32j**	**UJA34f**	**UJA35h**	**UJA37p**	**UJA40k**	**UJA40l**
**1**	^a^	^a^	^a^	10	10	^a^	^a^	10	50	^a^	^a^	^a^
**2**	50	50	50	50	^a^	^a^	^a^	50	10	^a^	50	10
**3**	^a^	^a^	^a^	10	10	^a^	^a^	10	50	^a^	^a^	^a^
**4**	50	50	50	50	10	50	50	50	10	100	50	10
**5**	^a^	^a^	^a^	10	10	^a^	^a^	10	10	^a^	^a^	^a^
**6**	^a^	10	10	10	50	^a^	^a^	^a^	50	^a^	^a^	10
**7**	^a^	^a^	^a^	10	10	^a^	^a^	10	10	^a^	^a^	^a^
**8**	50	10	50	10	10	50	50	10	10	100	50	10
						**(b)**						
**Analog**	***S. aureus*** **CECT 828**	***S. aureus*** **CECT 976**	***L. innocua*** **CECT 910**		***E. coli*** **CCUG47553**	***E. coli*** **CCUG47557**		***S. enterica*** **CECT 4300**	***S. enterica*** **CECT 409**		***S. enterica*** **CECT 4395**	***S. enterica*** **CECT 915**
**1**	50	50	^a^		^a^	^a^		^a^	^a^		^a^	^a^
**2**	10	10	50		50	^a^		^a^	^a^		^a^	50
**3**	50	50	^a^		^a^	^a^		^a^	^a^		^a^	^a^
**4**	10	10	10		100	100		100	100		100	50
**5**	50	50	50		^a^	^a^		^a^	^a^		^a^	^a^
**6**	10	10	10		^a^	^a^		^a^	^a^		^a^	^a^
**7**	50	50	50		^a^	^a^		^a^	^a^		^a^	^a^
**8**	10	10	10		100	100		^a^	100		^a^	100

^a^ MIC was above 1000 μg/mL. (a) Resistant strains from organic foods; (b) strains from type-culture collections.

The results of the antimicrobial activity obtained in these halogenated analogs to natural A-type proanthocyanidins overcame those previously found when a nitro group was added at the A-ring [7]. The results from this study also corroborated some structure–activity relationships previously described in nitro derivatives: those analogs with just one OH group at the B-ring (analogs **2**, **4**, **6** and **8**) showed better antimicrobial activity than those with two OH groups, regardless of having one or two OH groups at the D-ring or having chloro or bromo at the A-ring. In the same way, those analogs with two or three OH groups in their chemical formulae were more active than those with four OH groups. 

The checkerboard titer test was applied in order to find possible synergistic activities between the most active compounds (analogs **2**, **4**, **6** and **8**) against the most sensitive strains (*S. aureus* CECT 828 and CECT 976, *L. innocua* CECT 910, *S. saprophyticus* UJA 27g, *B. cereus* UJA 27q and *L. casei* UJA35h). Synergistic effects were found between analog **4** and analogs **2**, **6** and **8**, as well as between analogs **6** and **8**, against *S. aureus* CECT 828 (Table S1). Moreover, the same effect was observed between analogs **4** and **8** against *B. cereus* UJA 27q (Table S2). Indifferent results (with neither synergistic nor antagonistic effects) were obtained in the checkerboard assay against the other strains tested for any of the combinations assayed (Tables S3–S6).

### 2.4. Antibiofilm Activities

The analogs able to inhibit at least 75% of the formation and/or disrupt at least 75% of the established biofilms when compared to the control strains are shown in Table 4 and Table 5. Analogs **1** and **5** stood out for being able to block at least 75% of the formation of biofilm by ten of the highly resistant strains from organic foods, independent of the concentration tested. Analog **4** inhibited the formation of biofilm by three strains from organic foods and five strains from type-culture collections. Analogs **7** and **8** inhibited the formation of biofilm by seven strains, all from organic foods for analog **7** and also from type-culture collections in the last case. Finally, five strains were blocked in at least 75% of biofilm formation by analogs **2** and **3**. With respect to the disruption of the preformed biofilms, analogs **1**, **5** and **8** were able to disintegrate at least 75% of it in ten of the strains analyzed, including three strains from type-culture collections for analog **8**. The same effect was achieved by analog **7** on nine of the strains tested, and on five and three strains by analogs **4** and **3**, respectively.

When considering both anti-biofilm activities, analogs **1** and **5** stood out because of their high efficacy, so the combination of two OH groups at the B-ring and chloro at the A-ring seemed to potentiate these effects, especially at low concentrations. 

**Figure 2:**
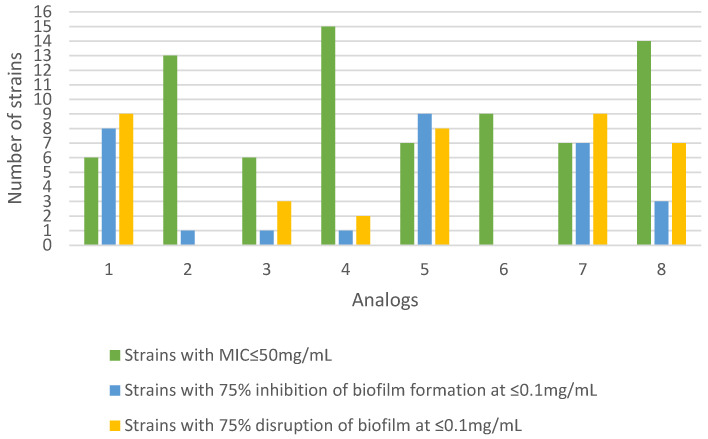
Key results of analogs **1**–**8** against target bacteria.

Figure 2 shows the key results of each of the studied analogs in both antimicrobial and antibiofilm activities, showing analogs **2**, **4** and **8** to have the best results on antimicrobial activity and analogs **1**, **5** and **7** to have the best antibiofilm activities.

The formation of biofilms is a successful strategy of bacteria to survive in the presence of biocides usually employed in food industries [3]. A particularly important problem in the food processing industry is those human pathogens able to form biofilm structures on different artificial substrates [11,12]. These biofilms usually increase food spoilage and disease outbreaks in many different food industries.

Modifying abiotic surface features, regulating signaling pathways and applying external substances are the main strategies for controlling biofilms in food industries [13,14,15]. However, tolerance to biocides usually employed in food industries is increasing, so new efficient compounds are necessary to address the rising challenge of bacterial resistance in food environments. In this study were described eight analogs to natural proanthocyanidins with high antimicrobial and antibiofilm activities against especially resistant bacteria from organic foods, which may constitute the basis of new suitable disinfectants and biocides for food production areas.

## 3. Materials and Methods

### 3.1. Chemicals and Instruments

All starting materials and reagents were purchased from Sigma-Aldrich (Steinheim, Germany) and Alfa Aesar (Karlsruhe, Germany). All solvents used in the chemical syntheses and preparative chromatographies were commercially available and used as received (Panreac, Spain) with the exception of absolute MeOH, which was previously dried according to reported methodologies [16]. Nuclear magnetic resonance (NMR) spectra of flavylium salts and analogues were recorded as reported in our previous paper in this series [7]. The following abbreviations are used for NMR signals: *d*, doublet; *t*, triplet; *m*, multiplet; *dd*, doublet of doublets; *ov*, overlapped signals. Analytical thin-layer chromatography (TLC), analytical high-performance liquid chromatography (HPLC) and semipreparative HPLC separations were performed as reported before [7]. High-resolution mass spectra (HRMS) were recorded on an Agilent 6520B Quadrupole time-of-flight (QTOF) mass spectrometer (Agilent Technologies, Santa Clara, CA, USA) with an electrospray ionization (ESI) interface operating in negative mode. Melting points (uncorrected) of all solids were measured on a Brastead Electrothermal 9100 apparatus (Electrothermal, Stone, Staffordshire, UK).

### 3.2. General Procedure A for the Synthesis of Flavylium Salts (***13***–***16***)

Aldehyde **9** or **10** (1 mmol) was mixed with the proper acetophenone derivative (**11** or **12**, 1 mmol). Then, 0.3 mL of 98% H_2_SO_4_ (5.4 mmol) and 1.3 mL of HOAc (1.3 mL) were added. The mixture was stirred overnight at room temperature following a similar procedure to that described by others [17]. Finally, 30 mL of Et_2_O was added to the mixture and a red solid precipitated. The solid formed was carefully filtered off, washed again with fresh Et_2_O and dried in a vacuum. All the synthetic flavylium prepared were previously prepared by us and their structure and purity were confirmed by a comparison of their physical and spectral data (^1^H NMR and ^13^C NMR) with those reported [8,9].

### 3.3. General Procedure B for the Synthesis of 2,8-Dioxabicyclo[3.3.1]nonane Derivatives (***1***–***8***)

A mixture of the flavylium salt derivative (**13**–**16**) and the nucleophilic moiety (phloroglucinol (**17**, 1 mmol) or resorcinol (**18**, 0.5 mmol)) was dissolved in 8 mL of absolute methanol. The mixture was stirred overnight at 50 °C using a similar procedure to that described by Kraus et al. [18]. Then, the solvent was removed, and the crude was purified by semipreparative HPLC or silica gel column chromatography (CC). Bicycle derivatives **1**–**5** and **7** were previously synthesized by us and their structure and purity were confirmed by a comparison of their physical and spectral data (^1^H NMR and ^13^C NMR) with those reported [8,9].

#### 3.3.1. 6-Chloro-2-(4′-hydroxyphenyl)-chromane-(4→4, 2→O-3)-resorcinol (**6**)

General procedure B was used with the flavylium salt **14** (0.171 g) and resorcinol (**18**, 0.055 g, 0.5 mmol). Then, the solvent was removed under vacuum and the final crude was purified by silica gel column chromatography (CC). The elution was performed with DCM-acetone (100:3) and pure analogue **6** was obtained as white solid (0.101 g, 36% from aldehyde **9**). Melting point: 230 °C (decomposes); ^1^H NMR (400 MHz, CD_3_OD) δ 7.41–7.36 (*m*, 2H, H-2′ (B), H-6′ (B)), 7.19 (*d*, *J* = 2.6, 1H, H-5(A)), 7.00 (*d*, *J* = 8.9, 1H, H-5″ (D)), 6.96 (*dd*, *J* = 8.6, 2.6, 1H, H-7(A)), 6.79 (*d*, *J* = 8.6, 1H, H-8(A)), 6.75–6.70 (*m*, 2H, H-3′ (B), H-5′ (B)), 6.28–6.26 (*ov*, 2H, H-2″(D), H-6″(D)), 3.91 (*d*, *J* = 3.3, 1H, H-4(C)), 2.23–2.15 (*m*, 2H, H-3(C)); ^13^C NMR (100 MHz, CD_3_OD) δ, 157.8 (C-4′ (B)), 157.4 (C-1″(D)), 152.7 (C-3″(D)), 150.9 (C-9(A)), 132.6 (C-1′ (B)), 129.6 (C-10(A)), 127.8 (C-6(A), C-5″(D)), 127.2 (C-7(A)), 126.8 (C-2′ (B), C-6′ (B)), 126.6 (C-5(A)), 117.5 (C-8(A), C-4″ (D)), 114.6 (C-3′ (B), C-5′ (B)), 108.8* (C-2″(D)), 102.9* (C-6″(D)), 98.9 (C-2(C)), 33.3 (C-4(C)), 33.2 (C-3(C)) (*these signals could be interchanged) (Figures S1–S4; Table S7); HRMS (ESI-TOF) *m*/*z* [M–H]^−^ calculated for C_21_H_14_ClO_4_ 365,0586, found 365,0655.

#### 3.3.2. 6-Bromo-2-(4′-hydroxyphenyl)-chromane-(4→4, 2→O-3)-resorcinol (**8**)

General procedure B was used with flavylium salt **16** (0.191 g) and resorcinol (**18**, 0.055 g, 0.5 mmol). Then, the solvent was removed under vacuum and the final crude was purified by silica gel column chromatography (CC). The elution was performed with DCM-acetone (100:3) and pure analogue **8** was obtained as a white solid (0.106 g, 37% from aldehyde **10**). Melting point: 230 °C (decomposes); ^1^H NMR (400 MHz, CD_3_OD) δ 7.40–7.35 (*m*, 2H, H-2′ (B), H-6′ (B)), 7.32 (*d*, *J* = 2.5, 1H, H-5(A)), 7.10 (*dd*, *J* = 8.6, 2.5, 1H, H-7(A)), 6.98 (*d*, *J* = 8.9, 1H, H-5″ (D)), 6.75–6.70 (*ov*, 3H, H-8(A), H-3′ (B), H-5′ (B)), 6.30–6.24 (*ov*, 2H, H-2″(D), H-6″(D)), 3.91 (*t*, *J* = 3.3, 1H, H-4(C)), 2.24–2.10 (*m*, 2H, H-3(C)); ^13^C NMR (100 MHz, CD_3_OD) δ, 159.2 (C-4′ (B)), 158.8 (C-1″(D)), 154.0 (C-3″(D)), 152.8 (C-9(A)), 133.9 (C-1′ (B)), 131.6 (C-7(A)), 131.5 (C-10(A)), 131.0 (C-5(A)), 129.2 (C-5″(D)), 128.2 (C-2′ (B), C-6′ (B)), 119.4 (C-8(A)), 118.9 (C-4″ (D)), 116.0 (C-3′ (B), C-5′ (B)), 114.2 (C-6(A)), 110.2* (C-2″(D)), 104.3* (C-6″(D)), 100.3 (C-2(C)), 34.6 (C-4(C)), 34.5 (C-3(C)) (*these signals could be interchanged) (Figures S5–S8; Table S8); HRMS (ESI-TOF) *m*/*z* [M–H]^−^ calculated for C_21_H_14_BrO_4_ 409,0081, found 409,0150.

The ^1^H NMR, ^13^C NMR and 2D NMR spectra of compounds **6** and **8** are shown in the Supplementary Materials.

### 3.4. DPPH Radical-Scavenging Activity

The antioxidant activity of analogs **1**–**8** and Trolox (used as a positive control) was determined as their radical-scavenging ability against the stable DPPH radical. This activity was spectrophotometrically measured on a Genesys^TM^ 150 Vis/UV-Vis spectrophotometer (Thermo Fischer Scientific, Waltham, MA, USA), conforming to a modified method of the one described by Brand-Williams [19] and von Gadow [20]. Briefly, methanolic solutions (1.2 mL) of DPPH (4.7 × 10^−5^ M), with an absorbance at 515 nm of 0.800 ± 0.030 AU, were mixed with methanolic solutions (0.6 mL) of samples at different concentrations (1–1000 ppm). The samples were shaken, and after 15 min in the dark at room temperature, the decrease of their absorbance at 515 nm was measured in triplicate. The radical-scavenging activity was expressed as the antioxidant concentration (µM) needed to decrease the initial DPPH^•^ concentration by 50% (Efficient Concentration: EC_50_). The percentage of the DPPH^•^ remaining was calculated by the following equation:

% DPPH_rem_ = [DPPH]/[DPPH]_0_ × 100 (1)

where [DPPH] is the concentration of DPPH^•^ at the time measured (t = 15 min), and [DPPH]_0_ is the initial concentration of DPPH^•^ (t = 0 min), plotted against the sample concentration (µg/mL). A linear regression could be established in order to calculate the EC_50_ (Table 1). A lower EC_50_ value indicates a stronger antioxidant activity in the sample.

### 3.5. Rancimat Assay

The antioxidant activity of analogs **1**–**8** and Cinnamtannin B-1 (C-B1; used as a positive control) was determined by the Rancimat methodology [21,22]. The activities were measured in a Rancimat 679 apparatus (Metrohm AG, Herisau, Switzerland) at 100 °C and an air flow rate of 20 L/h. The samples to be measured were prepared in the following manner: each analog or C-B1 (3 mg) was dissolved in MeOH (100 μL), refined soybean oil (Guinama SLU, Valencia, Spain) was added up to 10 g (300 ppm) and the whole was sonicated. Aliquots (3 g) of those samples were placed into the reaction vessels of the Rancimat device and the induction times (ITs) were measured. The relative activity of each analog (or C-B1) in the Rancimat assay is expressed by the protection factor (PF), which is calculated using the following equation:

PF = IT_inh_/IT_0_ (2)

where IT_inh_ is the induction time (in hours) of refined soybean oil with the analog (or C-B1) added, and IT_0_ is the induction time (in hours) of refined soybean oil alone. A longer IT (or a higher PF) value indicates a stronger antioxidant activity in the sample.

### 3.6. Antimicrobial Activity

The antimicrobial and antibiofilm activities of the analogs were evaluated in order to estimate their utility in food industries as biocides or food preservatives. All experiments were carried out in triplicate.

Preliminary studies on the antimicrobial activity of the compounds were performed by the standard agar diffusion method by dropping 5 μL of the different concentrations of the analogs, ranging from 1000 to 0.1 μg/mL, on Müller–Hinton agar plates previously seeded with the target bacteria. The diameters of inhibition zones were used to consider or not each of the compounds for further analysis. Next, minimal inhibitory concentration (MIC) values for each sample were determined by the broth microdilution method, according to the recommendations of the CLSI (2015) [23]. When the MIC for each target strain and analog was determined, the absence of growth after plating 100 μL from the wells onto nutrient agar plates was used to determine the minimum bactericidal concentration (MBC), which was the same as the MIC in all cases. Strains from the Spanish Type-Culture Collection (CECT), the Culture Collection of the University of Goteborg (CCUG) as well as strains from our own collection from organic foods (Table 6), were used as targets for these assays. Genetic identification at the species level was performed by PCR analysis with species–specific primers for enterococci and by 16S rRNA sequencing for the rest of the isolates, as described in [24].

### 3.7. Checkerboard Titer Tests

The checkerboard method was used in order to search for possible synergistic effects between the most active analogs against food pathogens. These results are expressed as the sum of the fractional inhibitory concentration (FIC) index for each agent (fractional number resulting from the MIC of an agent in combination divided by the MIC of this compound alone). The FIC value of the most effective combination was used in calculating the fractional inhibitory concentration index (FICI) by adding both FICs: FICI = FICA + FICB = CAcomb/MICAalone + CBcomb/MICBalone, where MICAalone and MICBalone are the MICs of drugs A and B when acting alone, and CAcomb and CBcomb are the concentrations of drugs A and B at the isoeffective combinations, respectively. The FICI was interpreted as a synergistic activity when it was ≤0.5, antagonistic when it was >4.0 and any value in between was interpreted as indifferent, according to Rukayadi et al., 2009 [25] and Guo et al., 2010 [26].

### 3.8. Biofilm Formation Inhibition Assay

In order to detect the positive effects of these analogs on inhibiting the formation of biofilms by the target strains, bacteria were incubated with 10-fold serially diluted purified analogs, ranging from 0.1 μg/mL to 10 μg/mL, depending on the MIC values previously obtained, according to Ulrey et al. [27]. The inhibition of biofilm formation was detected by the crystal violet stain method as we had previously described [6].

### 3.9. Disruption of Preformed Biofilm

In order to study the ability of the analogs in disrupting previously formed biofilms by food pathogens, cells were allowed to settle biofilms during 24 h, and once the bacteria had expanded these structures, appropriate diluted compounds were added to the plates, and after a second incubation (24 h, 30 °C), the remaining biofilm was measured by the crystal violet stain method, as previously described.

### 3.10. Statistical Analysis

The average data and standard deviations from absorbances were determined with the Excel program version 18.0 (Microsoft Corp., Redmond, WA, USA). A *t*-test was performed at the 95% confidence level with Statgraphics Plus version 5.1 (Statistical Graphics Corp., Rockville, MD, USA) to determine the statistical significance of the data.

## 4. Conclusions

The results from this study show that halogenated analogs to natural A-type proanthocyanidins rose above the nitro derivatives previously reported [7] in their antimicrobial activities. Gram-positive bacteria were the most sensitive to all the analogs and combinations assayed, showing MICs from 10 to 50 μg/mL in most cases, as well as reductions of at least 75% of biofilm formation as well as the disruption of preformed biofilms. Some structure–activity relationships previously described for nitro analogs were also corroborated when studying these halogenated compounds, so those analogs with just one OH group at the B-ring (**2**, **4**, **6**, **8**) showed better antimicrobial activities than those with two OH groups (**1**, **3**, **5**, **7**), and those analogs with two or three OH groups in their whole structure (**2**, **4**–**8**) were more active than those with four OH groups in total (**1**, **3**). In contrast, the analogs with two OH groups at the B-ring and chloro at the A-ring (**1**, **5**) were the most effective ones when antibiofilm activities were studied, especially at low concentrations. Regarding the antioxidant activity, analogs with a catechol moiety at the B-ring (**1**, **3**, **5**, **7**) had higher radical-scavenging and oxidative stability activities than analogs without it (**2**, **4**, **6**, **8**). This means that antimicrobial and antioxidant activities pointed in opposite directions. However, the analogs with better antibiofilm activities (**1**, **5**) were two of the four most active compounds in terms of antioxidant capacity. Taking all these results into consideration, future assays in collaboration with the food packaging industry are being planned in order to improve the efficacy of their tray and plastic films for food preservation. 

## Figures and Tables

**Table 1:** Antioxidant activity of analogs **1**–**8** estimated as efficient concentration (EC_50_) and protection factor (PF) ^a^.

Compound	EC_50_ ^b^	PF ^c^
**1**	0.29 ± 0.01	1.18 ± 0.01
**2**	5.06 ± 0.19	0.92± 0.01
**3**	0.30 ± 0.02	1.25 ± 0.02
**4**	6.58 ± 0.08	1.06 ± 0.01
**5**	0.31 ± 0.01	1.31 ± 0.02
**6**	>12	1.03 ± 0.01
**7**	0.30 ± 0.01	1.19 ± 0.01
**8**	>12	1.01 ± 0.01
Trolox ^d^	0.24 ± 0.01	-
Cinnamtannin B-1 (C-B1) ^d^	-	1.06 ± 0.01

^a^ Data are expressed as means of three determinations ± SD. ^b^ Efficient concentration (EC_50_) values in the DPPH radical-scavenging activity assay, expressed as mmol of compound/mmol DPPH. ^c^ Protection factor (PF) in the Rancimat assay, expressed as induction time (IT) of refined soybean oil supplemented with a compound divided by the IT of refined soybean oil alone. ^d^ Antioxidant used as a reference.

**Table 4:** Inhibition of biofilm formation by analogs **1–8** against resistant strains from organic foods and strains from type-culture collections.

Analog	Inhibition of Biofilm Formation of at Least 75%
**1**	UJA7m (10 µg/mL, 0.01 µg/mL) UJA11c (1 µg/mL, 0.01 µg/mL) UJA11e (10 µg/mL, 1 µg/mL, 0.1 µg/mL, 0.01 µg/mL) UJA27q (10 µg/mL, 1 µg/mL, 0.1 µg/mL, 0.01 µg/mL) UJA29o (0.1 µg/mL, 0.01 µg/mL) UJA32j (10 µg/mL, 1 µg/mL, 0.1 µg/mL, 0.01 µg/mL) UJA34f (10 µg/mL, 1 µg/mL, 0.1 µg/mL, 0.01 µg/mL) UJA35h (10 µg/mL) UJA37p (10 µg/mL) UJA40k (10 µg/mL, 1 µg/mL, 0.01 µg/mL)
**2**	UJA7m (1 µg/mL) UJA34f (1 µg/mL) *Salmonella enterica* CECT 915 (0.001 µg/mL) *Escherichia coli* CCUG 47553 (1 µg/mL) *Listeria innocua* CECT 910 (1 µg/mL)
**3**	UJA7m (10 µg/mL) UJA32j (10 µg/mL) UJA34f (10 µg/mL, 1 µg/mL) UJA35h (0.01 µg/mL) UJA40l (10 µg/mL)
**4**	UJA7m (1 µg/mL) UJA27g (10 µg/mL) UJA27q (10 µg/mL) *Staphylococcus aureus* CECT 828 (0.001 µg/mL) *Escherichia coli* CCUG 47557 (10 µg/mL) *Salmonella enterica* CECT 4300 (10 µg/mL) *Salmonella enterica* CECT 409 (50 µg/mL) *Escherichia coli* CCUG 47553 (50 µg/mL)
**5**	UJA11c (10 µg/mL, 0.01 µg/mL) UJA11e (10 µg/mL, 0.1 µg/mL, 0.01 µg/mL) UJA27g (10 µg/mL, 0.01 µg/mL) UJA27q (10 µg/mL, 0.01 µg/mL) UJA32j (10 µg/mL, 1 µg/mL, 0.1 µg/mL) UJA34f (10 µg/mL, 1 µg/mL, 0.1 µg/mL, 0.01 µg/mL) UJA35h (0.1 µg/mL, 0.01 µg/mL) UJA37p (10 µg/mL, 1 µg/mL, 0.1 µg/mL) UJA40k (1 µg/mL) UJA40l (10 µg/mL, 1 µg/mL, 0.1 µg/mL, 0.01 µg/mL)
**7**	UJA7m (0.1 µg/mL, 0.01 µg/mL) UJA11c (0.1 µg/mL, 0.01 µg/mL) UJA11e (10 µg/mL, 0.1 µg/mL, 0.01 µg/mL) UJA27g (10 µg/mL, 1 µg/mL, 0.1 µg/mL, 0.01 µg/mL) UJA29o (10 µg/mL, 1 µg/mL, 0.1 µg/mL) UJA32j (10 µg/mL, 0.1 µg/mL) UJA37p (1 µg/mL, 0.1 µg/mL)
**8**	UJA7m (10 µg/mL, 1 µg/mL, 0.1 µg/mL) UJA11e (10 µg/mL) UJA29o (50 µg/mL, 0.1 µg/mL) UJA32j (0.01 µg/mL) *Salmonella enterica* CECT 915 (10 µg/mL) *Escherichia coli* CCUG 47553 (50 µg/mL) *Escherichia coli* CCUG 47557 (10 µg/mL)

**Table 5:** Disruption of preformed biofilm by analogs **1–8** against resistant strains from organic foods and strains from type-culture collections.

Analog	Disruption of at Least 75% of Preformed Biofilms
**1**	UJA7m (10 µg/mL) UJA11c (0.1 µg/mL, 0.01 µg/mL) UJA11e (1 µg/mL, 0.1 µg/mL, 0.01 µg/mL) UJA27g (10 µg/mL, 1 µg/mL, 0.01 µg/mL) UJA29o (10 µg/mL, 0.1 µg/mL) UJA32j (0.01 µg/mL) UJA35h (0.1 µg/mL, 0.01 µg/mL) UJA37p (0.1 µg/mL, 0.01 µg/mL) UJA40k (0.1 µg/mL) UJA40l (10 µg/mL, 1 µg/mL, 0.1 µg/mL, 0.01 µg/mL)
**3**	UJA7m (0.01 µg/mL) UJA11e (0.1 µg/mL, 0.01 µg/mL) UJA32j (0.01 µg/mL)
**4**	UJA7m (10 µg/mL) UJA27q (1 µg/mL, 0.01 µg/mL) *Staphylococcus aureus* CECT 828 (1 µg/mL, 0.01 µg/mL, 0.001 µg/mL) *Staphylococcus aureus* CECT 976 (1 µg/mL) *Salmonella enterica* CECT 915 (10 µg/mL, 1 µg/mL)
**5**	UJA7m (0.01 µg/mL) UJA11c (0.01 µg/mL) UJA11e (0.01 µg/mL) UJA27q (10 µg/mL) UJA32j (10 µg/mL, 1 µg/mL) UJA34f (0.1 µg/mL, 0.01 µg/mL) UJA35h (1 µg/mL, 0.01 µg/mL) UJA37p (10 µg/mL, 1 µg/mL, 0.1 µg/mL, 0.01 µg/mL) UJA40k (1 µg/mL, 0.01 µg/mL) UJA40l (1 µg/mL, 0.1 µg/mL, 0.01 µg/mL)
**7**	UJA7m (0.01 µg/mL) UJA11c (10 µg/mL, 0.01 µg/mL) UJA11e (0.01 µg/mL) UJA27g (0.1 µg/mL) UJA29o (0.1 µg/mL) UJA32j (0.1 µg/mL) UJA34f (10 µg/mL, 0.1 µg/mL,) UJA37p (10 µg/mL, 1 µg/mL, 0.1 µg/mL, 0.01 µg/mL) UJA40l (10 µg/mL, 1 µg/mL, 0.1 µg/mL, 0.01 µg/mL)
**8**	UJA7m (10 µg/mL, 1 µg/mL, 0.01 µg/mL) UJA11c (0.001 µg/mL) UJA11e (10 µg/mL) UJA27q (0.001 µg/mL) UJA29o (10 µg/mL) UJA34f (1 µg/mL, 0.1 µg/mL, 0.001 µg/mL) UJA35h (1 µg/mL, 0.1 µg/mL, 0.01 µg/mL, 0.001 µg/mL) *Staphylococcus aureus* CECT 828 (0.001 µg/mL) *Escherichia coli* CCUG 47553 (50 µg/mL) *Listeria innocua* CECT 910 (0.001 µg/mL)

**Table 6:** Bacterial strains tested in antimicrobial assays.

Strains from Type-Culture Collections	Resistant Strains from Organic Foods
*Salmonella enterica* CECT 915	*Bacillus cereus* UJA27q
*Salmonella enterica* CECT 4300	*Enterococcus casseliflavus* UJA11e
*Escherichia coli* CCUG 47553	*Enterococcus faecium* UJA11c
*Escherichia coli* CCUG 47557	*Staphylococcus aureus* UJA34f
*Staphylococcus aureus* CECT 828	*Staphylococcus saprophyticus* UJA27g
*Staphylococcus aureus* CECT 976	*Lactobacillus casei* UJA35h
*Staphylococcus aureus* CECT 4465	*Enterobacter* sp. UJA37p
*Listeria monocytogenes* CECT 4032	*Pantoea agglomerans* UJA7m
	*Pantoea agglomerans* UJA29o
	*Klebsiella terrigena* UJA32j
	*Salmonella* sp. UJA40k
	*Salmonella* sp. UJA40l

## Data Availability

Data are contained within the article and Supplementary Materials.

## Supplementary Materials

The following supporting information can be downloaded at: https: https://www.mdpi.com/article/10.3390/molecules29153622/s1, Experimental procedures to synthesize already known compounds [9]; Table S1: Checkerboard assay of analogs against *Staphylococcus aureus* CECT 828; Table S2: Checkerboard assay of analogs against *Bacillus cereus* UJA27q; Table S3: Checkerboard assay of analogs against *Staphylococcus aureus* CECT 976; Table S4: Checkerboard assay of analogs against *Listeria innocua* CECT 910; Table S5: Checkerboard assay of analogs against *Staphylococcus saprophyticus* UJA27g; Table S6: Checkerboard assay of analogs against *Lactobacillus casei* UJA35h; Figure S1: ^1^H-NMR spectrum of analog **6** in CD_3_OD; Figure S1a: ^1^H-NMR spectrum of analog **6** in CD_3_OD (7.4–6.1 ppm ampliation); Figure S2: ^13^C-NMR spectrum of analog **6** in CD_3_OD; Figure S2a: ^13^C-NMR spectrum of analog **6** in CD_3_OD (160–96 ppm ampliation); Figure S3: ^1^H-^13^C-HMBC spectrum of analog **6** in CD_3_OD; Figure S4: ^1^H-^1^H-COSY spectrum of analog **6** in CD_3_OD; Table S7: ^1^H-NMR and ^13^C-NMR full peak assignment of analog **6** in CD_3_OD; Figure S5: ^1^H-NMR spectrum of analog **8** in CD_3_OD. Figure S5a: ^1^H-NMR spectrum of analog **8** in CD_3_OD. (7.5–6.2 ppm ampliation); Figure S6: ^13^C-NMR spectrum of analog **8** in CD_3_OD; Figure S6a: ^13^C-NMR spectrum of analog **8** in CD_3_OD (160–96 ppm ampliation); Figure S7: ^1^H-^13^C-HMBC spectrum of analog **8** in CD_3_OD; Figure S8: ^1^H-^1^H-COSY spectrum of analog **8** in CD_3_OD; Table S8: ^1^H-NMR and ^13^C-NMR full peak assignment of analog **8** in CD_3_OD.
