# Supplementary material for: Halogenated Analogs to Natural A-Type Proanthocyanidins: Evaluation of Their Antioxidant and Antimicrobial Properties and Possible Application in Food Industries
Source: Molecules. 2024 Jul 31;29(15):3622. doi: 10.3390/molecules29153622 (PMC11314616; doi:10.3390/molecules29153622)
Supplement: Supplementary file 1 [file molecules-29-03622-s001.zip › molecules-3098845-supplementary.pdf]

SUPPLEMENTARY MATERIALS

Halogenated Analogs to Natural A-Type Proanthocyanidins: Evaluation of Their Antioxidant and Antimicrobial Properties and Possible Application in Food Industries

Antonio Cobo [1,†], Alfonso Alejo-Armijo [2], Daniel Cruz [1], Joaquín Altarejos [2], Sofía Salido [2,*] and Elena Ortega-Morente [1,*]

Experimental procedures to synthesize already known compounds [Ref. 9]:

6-Chloro-3',4'-dihydroxyflavylium hydrogen sulfate (**13**).

General procedure A was followed by using aldehyde **9** (0.157 g, 1 mmol) and 3',4'-dihydroxyacetophenone (**11**) (0.152 g, 1 mmol). A red-brownish solid was obtained by treating the solution with Et_2O. The solid was filtered off, washed with Et_2O, and dried, yielding the expected compound **13** (0.304 g).

6-Chloro-4'-hydroxyflavylium hydrogen sulfate (**14**).

General procedure A was followed by using aldehyde **9** (0.157 g, 1 mmol) and 4'-hydroxyacetophenone (**12**) (0.136 g, 1 mmol). A red-orange solid was obtained by treating the solution with Et_2O. The solid was filtered off, washed with Et_2O, and dried, yielding the expected compound **14** (0.224 g).

6-Bromo-3',4'-dihydroxyflavylium hydrogen sulfate (**15**).

General procedure A was followed by using aldehyde **10** (0.201 g, 1 mmol) and 3',4'-dihydroxyacetophenone (**11**) (0.152 g, 1 mmol). A red-brownish solid was obtained by treating the solution with Et_2O. The solid was filtered off, washed with Et_2O, and dried, yielding the expected compound **15** (0.372 g).

6-Bromo-4'-hydroxyflavylium hydrogen sulfate (**16**).

General procedure A was followed by using aldehyde **10** (0.201 g, 1 mmol) and 4'-hydroxyacetophenone (**12**) (0.136 g, 1 mmol). A red-orange solid was obtained by treating the solution with Et_2O. The solid was filtered off, washed with Et_2O, and dried, yielding the expected compound **16** (0.273 g).

6-Chloro-2-(3',4'-dihydroxyphenyl)-chromane-(4→4,2→O-5)-phloroglucinol (**1**).

General procedure B was used with the flavylium salt **13** (0.185 g) and phloroglucinol (**17**, 0.126 g, 1 mmol). Then, the solvent was removed under vacuum and the final crude was purified by silica gel column chromatography (CC). The elution was performed with DCM-EtOH (97:3) and pure analogue **1** was obtained as colorless foam (0.084 g, 43% from aldehyde **9**).

6-Chloro-2-(4'-hydroxyphenyl)-chromane-(4→4,2→O-5)-phloroglucinol (**2**).

General procedure B was used with the flavylium salt **14** (0.171 g) and phloroglucinol (**17**, 0.126 g, 1 mmol). Then, the solvent was removed under vacuum and the final crude was purified by

semipreparative HPLC with MeOH-H_2O (60:40). Pure analogue **2** was obtained as a colorless foam (0.077 g, 42% from aldehyde **9**).

6-Bromo-2-(3',4'-dihydroxyphenyl)-chromane-(4→4,2→O-5)-phloroglucinol (**3**).

General procedure B was used with the flavylium salt **15** (0.208 g) and phloroglucinol (**17**, 0.126 g, 1 mmol). Then, the solvent was removed under vacuum and the final crude was purified by semipreparative HPLC with MeOH-H_2O (60:40). Pure analogue **3** was obtained as a brown syrup (0.096 g, 41% from aldehyde **10**).

6-Bromo-2-(4'-hydroxyphenyl)-chromane-(4→4,2→O-5)-phloroglucinol (**4**).

General procedure B was used with the flavylium salt **16** (0.191 g) and phloroglucinol (**17**, 0.126 g, 1 mmol). Then, the solvent was removed under vacuum and the final crude was purified by semipreparative HPLC with MeOH-H_2O (60:40). Pure analogue **4** was obtained as a colorless foam (0.100 g, 49% from aldehyde **10**).

6-Chloro-2-(3',4'-dihydroxyphenyl)-chromane-(4→4,2→O-3)-resorcinol (**5**).

General procedure B was used with the flavylium salt **13** (0.185 g) and resorcinol (**18**, 0.055 g, 0.5 mmol). Then, the solvent was removed under vacuum and the final crude was purified by semipreparative HPLC with MeOH-H_2O (60:40). Pure analogue **5** was obtained as a brown syrup (0.065 g, 36% from aldehyde **9**).

6-Bromo-2-(3',4'-dihydroxyphenyl)-chromane-(4→4,2→O-3)-resorcinol (**7**).

General procedure B was used with the flavylium salt **15** (0.208 g) and resorcinol (**18**, 0.055 g, 0.5 mmol). Then, the solvent was removed under vacuum and the final crude was purified by semipreparative HPLC with MeOH-H_2O (60:40). Pure analogue **7** was obtained as a brown reddish syrup (0.054 g, 35% from aldehyde **10**).

Table S1. Checkerboard assay of analogs against *Staphylococcus aureus* CECT 828.

	MIC of each agent (µg/mL)				
**Analog**	**Alone**	**Combination**	**FIC**	**FICI**	**Outcome**
**2**	10	5	0.5		
**6**	10	5	0.5	1	IND
**2**	10	2.5	0.25		
**4**	10	2.5	0.25	0.5	SYN
**2**	10	5	0.5		
**8**	10	1.25	0.125	0.625	IND
**6**	10	2.5	0.25		
**4**	10	2.5	0.25	0.5	SYN
**6**	10	1.25	0.125		
**8**	10	2.5	0.25	0.375	SYN
**4**	10	2.5	0.25		
**8**	10	2.5	0.25	0.5	SYN

MIC: minimal inhibitory concentration; FIC: fractional inhibitory concentration (FIC=MIC combination/MIC alone); FICI= FIC of one compound + FIC of the other compound.

SYN: synergy. IND: indifferent.

Table S2. Checkerboard assay of analogs against *Bacillus cereus* UJA27q.

	MIC of each agent (µg/mL)				
**Analog**	**Alone**	**Combination**	**FIC**	**FICI**	**Outcome**
**4**	10	2.5	0.25		
**8**	10	2.5	0.25	0.5	SYN

MIC: minimal inhibitory concentration; FIC: fractional inhibitory concentration (FIC=MIC combination/MIC alone); FICI= FIC of one compound + FIC of the other compound.

SYN: synergy. IND: indifferent.

Table S3. Checkerboard assay of analogs against *Staphylococcus aureus* CECT 976.

	MIC of each agent (µg/mL)				
**Analog**	**Alone**	**Combination**	**FIC**	**FICI**	**Outcome**
**2**	10	5	0.5		
**6**	10	5	0.5	1	IND
**2**	10	1.25	0.125		
**4**	10	10	1	1.125	IND
**2**	10	10	1		
**8**	10	1.25	0.125	1.125	IND
**6**	10	10	1		
**4**	10	1.25	0.125	1.125	IND
**6**	10	1.25	0.125		
**8**	10	5	0.5	0.625	IND
**4**	10	2.5	0.25		
**8**	10	5	0.5	0.75	IND

MIC: minimal inhibitory concentration; FIC: fractional inhibitory concentration (FIC=MIC combination/MIC alone); FICI= FIC of one compound + FIC of the other compound.

SYN: synergy. IND: indifferent.

Table S4. Checkerboard assay of analogs against *Listeria innocua* CECT 910.

	MIC of each agent (µg/mL)				
**Analog**	**Alone**	**Combination**	**FIC**	**FICI**	**Outcome**
**6**	10	10	1		
**4**	10	1.25	0.125	1.125	IND
**6**	10	5	0.5		
**8**	10	1.25	0.125	0.625	IND
**4**	10	1.25	0.125		
**8**	10	0.5	0.5	0.625	IND

MIC: minimal inhibitory concentration; FIC: fractional inhibitory concentration (FIC=MIC combination/MIC alone); FICI= FIC of one compound + FIC of the other compound.

SYN: synergy. IND: indifferent.

Table S5. Checkerboard assay of analogs against *Staphylococcus saprophyticus* UJA27g.

	MIC of each agent (µg/mL)				
**Analog**	**Alone**	**Combination**	**FIC**	**FICI**	**Outcome**
**6**	10	10	1		
**8**	10	10	1	2	IND

MIC: minimal inhibitory concentration; FIC: fractional inhibitory concentration (FIC=MIC combination/MIC alone); FICI= FIC of one compound + FIC of the other compound.

SYN: synergy. IND: indifferent.

Table S6. Checkerboard assay of analogs against *Lactobacillus casei* UJA35h.

	MIC of each agent (µg/mL)				
**Analog**	**Alone**	**Combination**	**FIC**	**FICI**	**Outcome**
**2**	10	5	0.5		
**4**	10	1.25	0.125	0.625	IND
**2**	10	5	0.5		
**8**	10	1.25	0.125	0.625	IND
**4**	10	1.25	0.125		
**8**	10	5	0.5	0.625	IND

MIC: minimal inhibitory concentration; FIC: fractional inhibitory concentration (FIC=MIC combination/MIC alone); FICI= FIC of one compound + FIC of the other compound.

SYN: synergy. IND: indifferent.

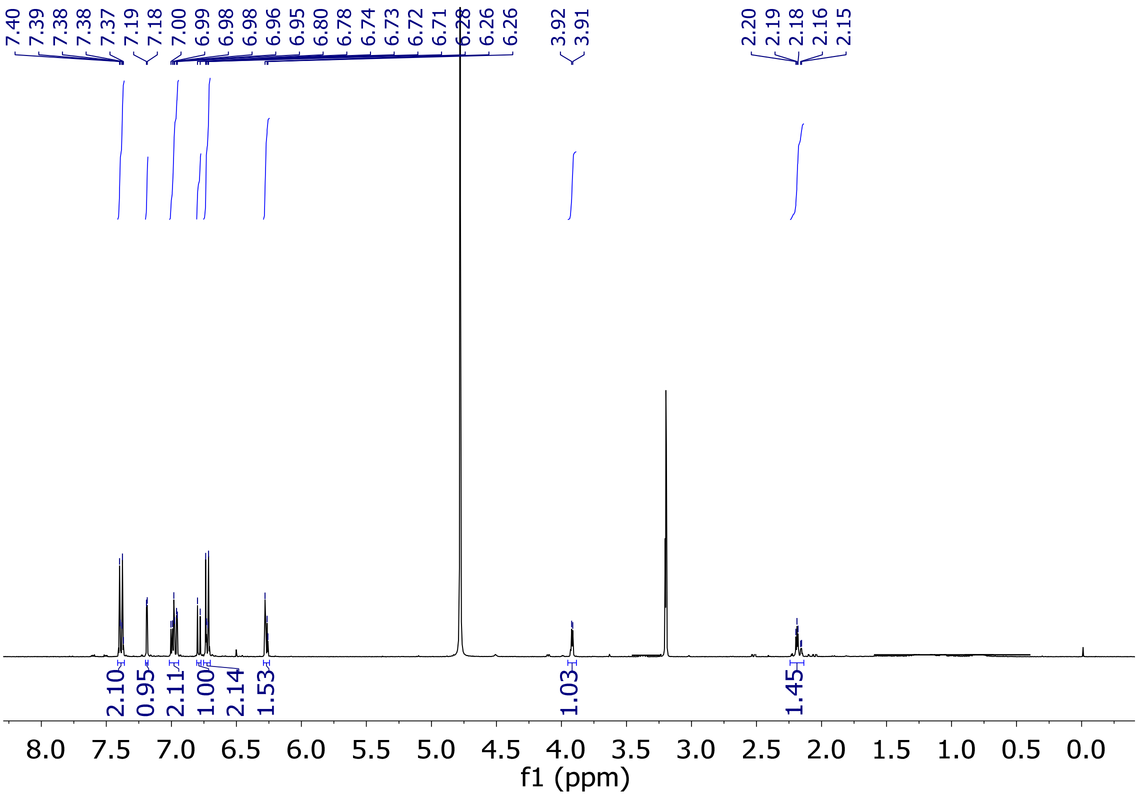

Figure S1. ^{1}H-NMR spectrum of analog **6** in CD_3OD.

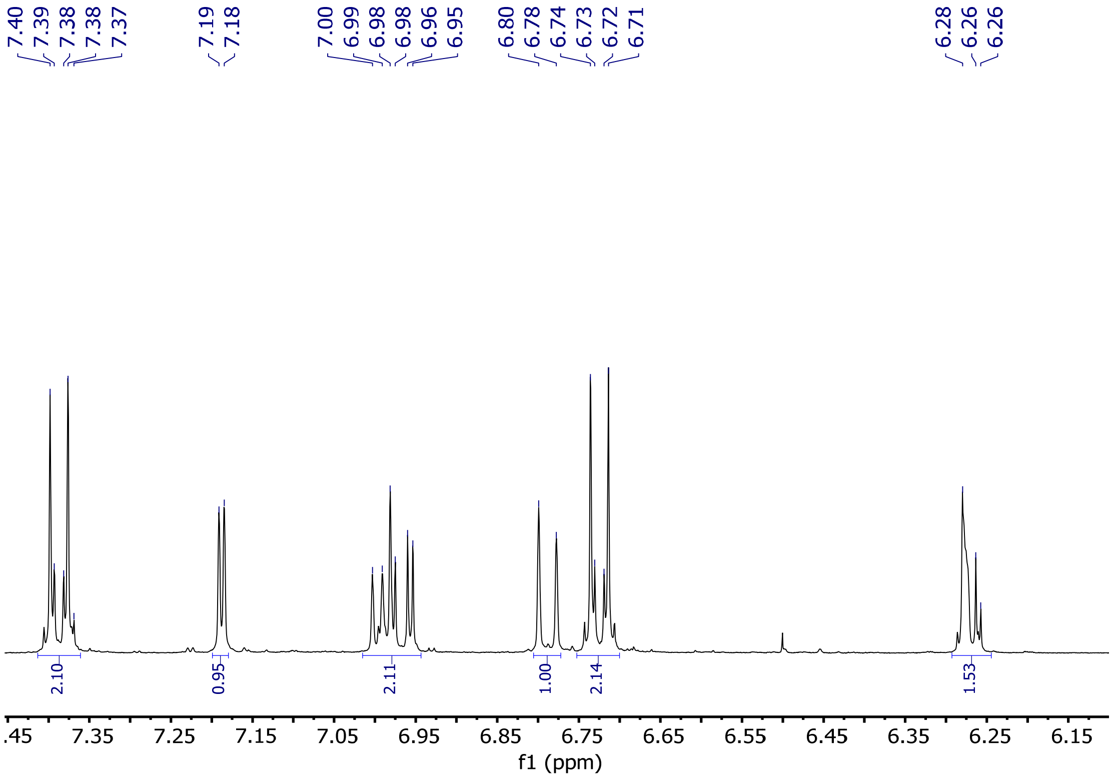

Figure S1a. ^{1}H-NMR spectrum of analog **6** in CD_3OD (7.4–6.1ppm ampliation).

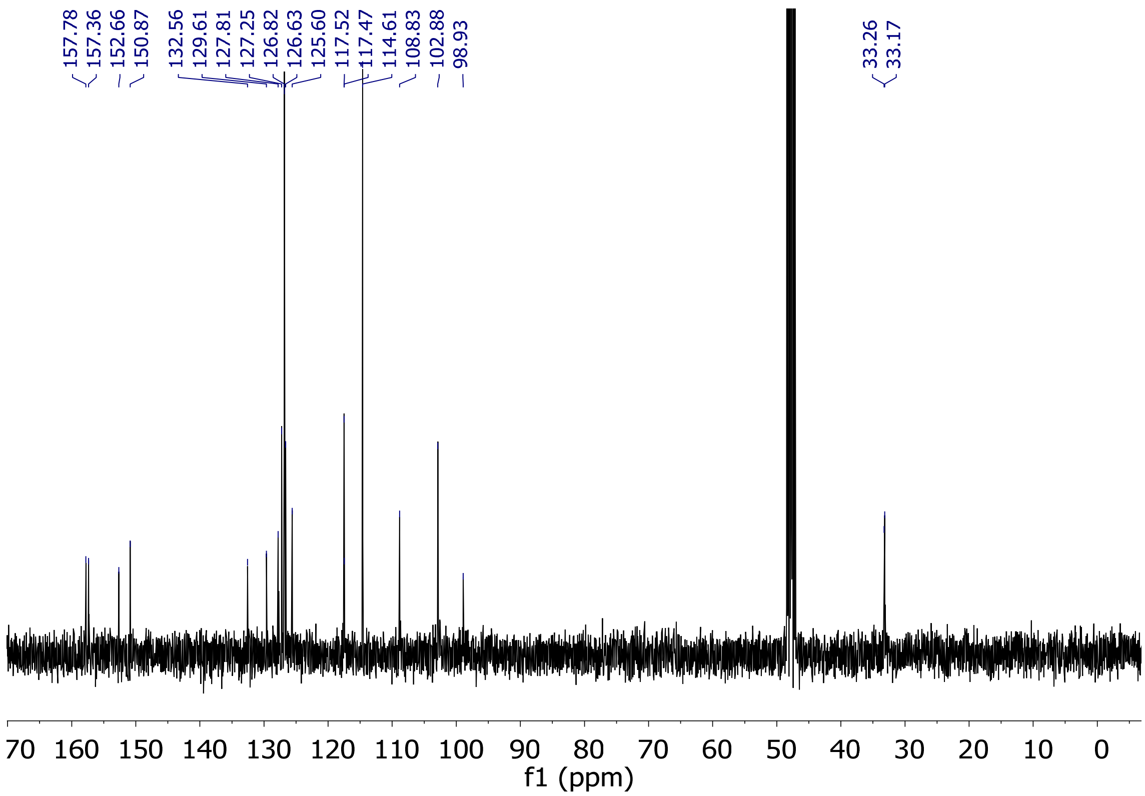

Figure S2. ^{13}C-NMR spectrum of analog **6** in CD_3OD.

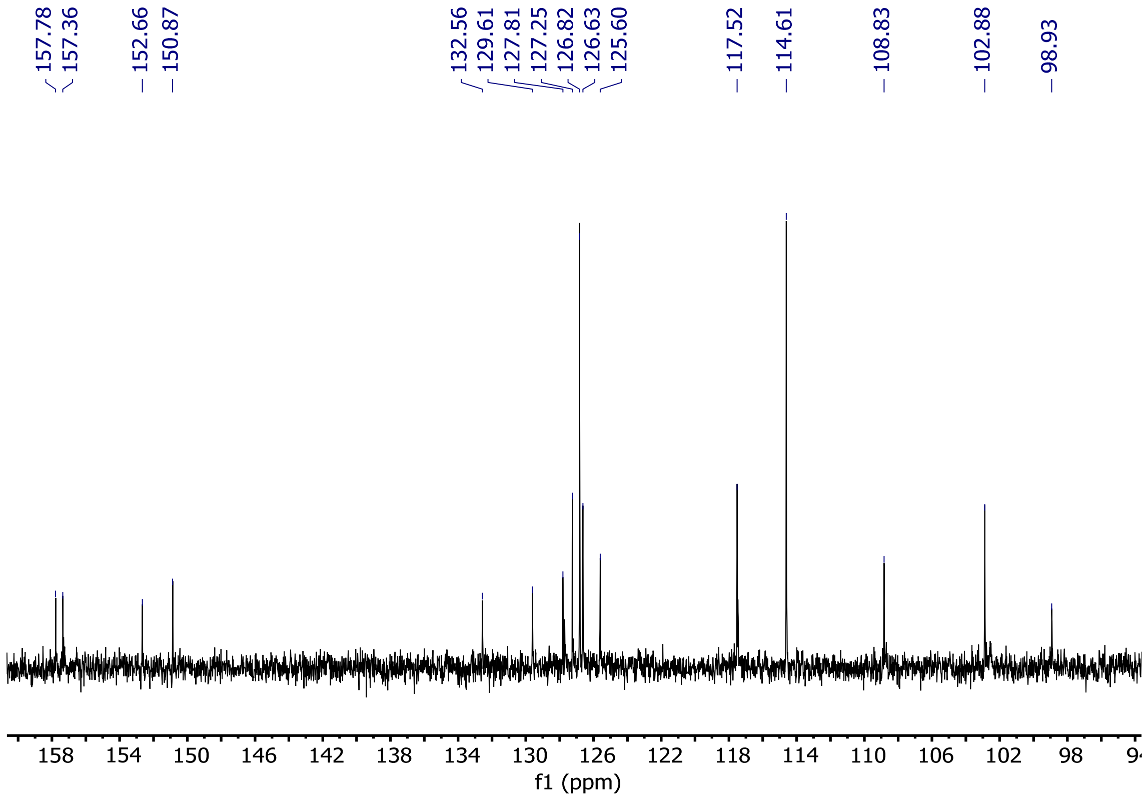

Figure S2a. ^{13}C-NMR spectrum of analog **6** in CD_3OD (160–96 ppm ampliation).

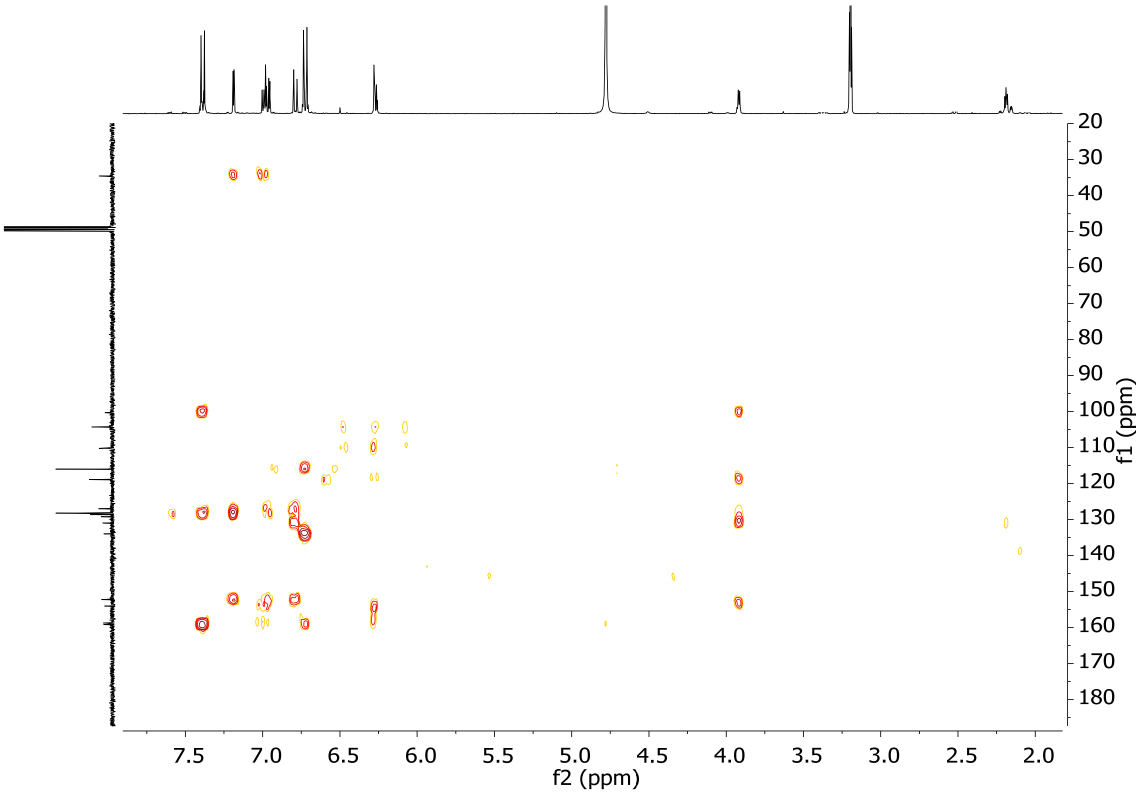

Figure S3. ^{1}H-^{13}C-HMBC spectrum of analog **6** in CD_3OD.

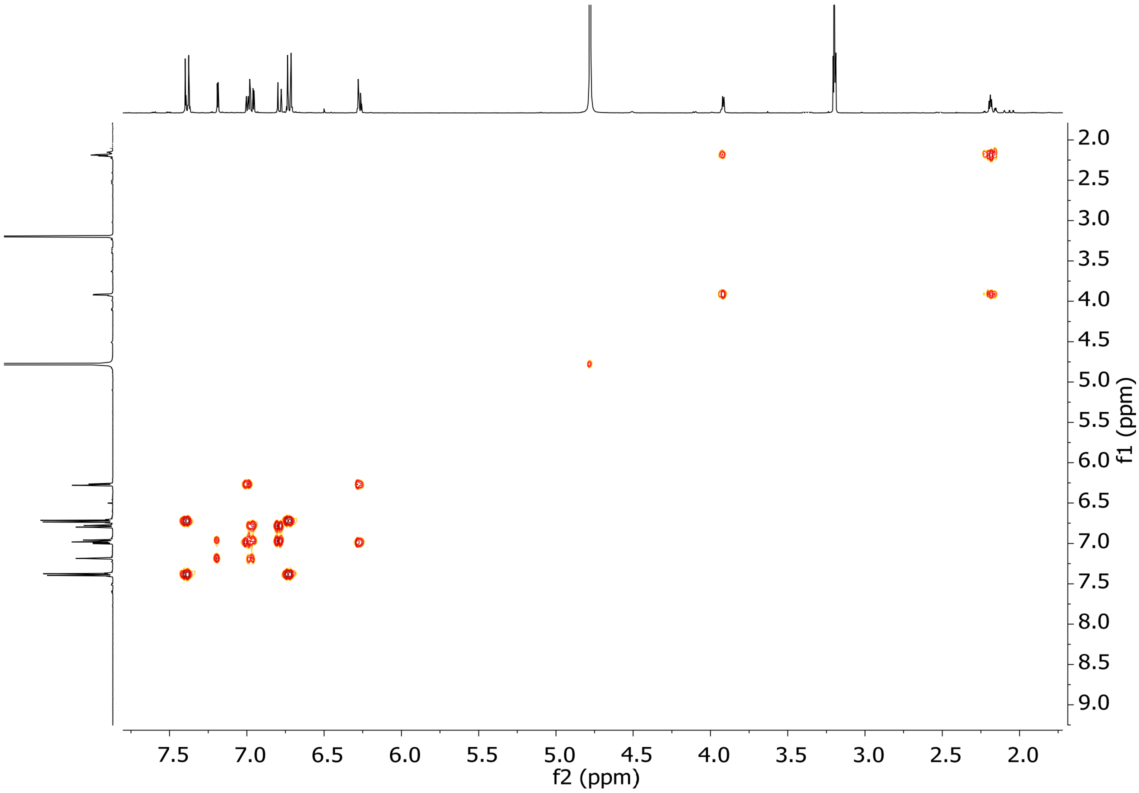

Figure S4. ^{1}H-^{1}H-COSY spectrum of analog **6** in CD_3OD.

Table S7. ^{1}H-NMR and ^{13}C-NMR full peak assignment of analog **6** in CD_3OD.

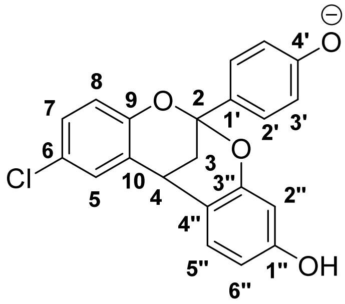

Position[a]	^{1}H /ppm (J/Hz) MeOD	^{13}C /ppm MeOD	HMBC MeOD
1	-	-	-
2	-	98.9	2, 2′, 3, 4
3	2.15-2.23 (*m*)	33.2	2, 10, 4″
4	3.91(*d*, *J* = 3.3 Hz)	33.3	5, 9, 3″, 5″
5	7.19(*d*, *J* = 2.6 Hz)	126.6	4, 7, 9
6	-	125.6	8
7	6.96 (*dd*, *J* = 8.6,2.6 Hz)	127.2	5, 9
8	6.79 (*d*, *J* = 8.6 Hz)	117.5	6, 10
9	-	150.9	4, 5, 7
10	-	129.6	3, 8
1′	-	132.6	3′
2′	7.36-7.41 (*m*)	126.8	2, 4′
3′	6.70-6.75 (*m*)-	114.6	1′
4′	-	157.8	2′
1″	-	157.4	5″
2″	6.26-6.28 (*ov*)	108.8*	4″, 6″
3″	-	152.7	4, 5″
4″	-	117.5	3, 6″
5″	7.00 (*d*, *J* = 8.9 Hz)	127.8	1″, 3″, 4
6″	6.26-6.28 (*ov*)	102.9*	2″, 4″

[a]Numbering was done according to the flavylium structure. * these signals could be interchangeable.

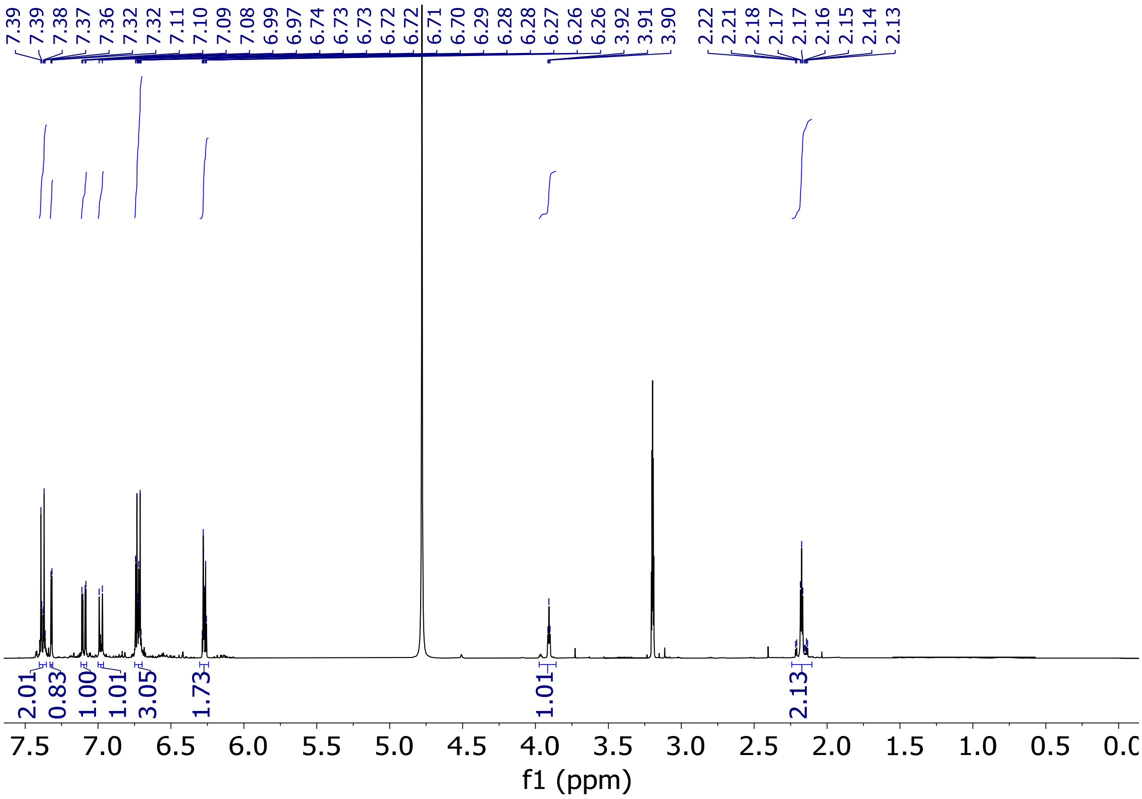

Figure S5. ^{1}H-NMR spectrum of analog **8** in CD_3OD.

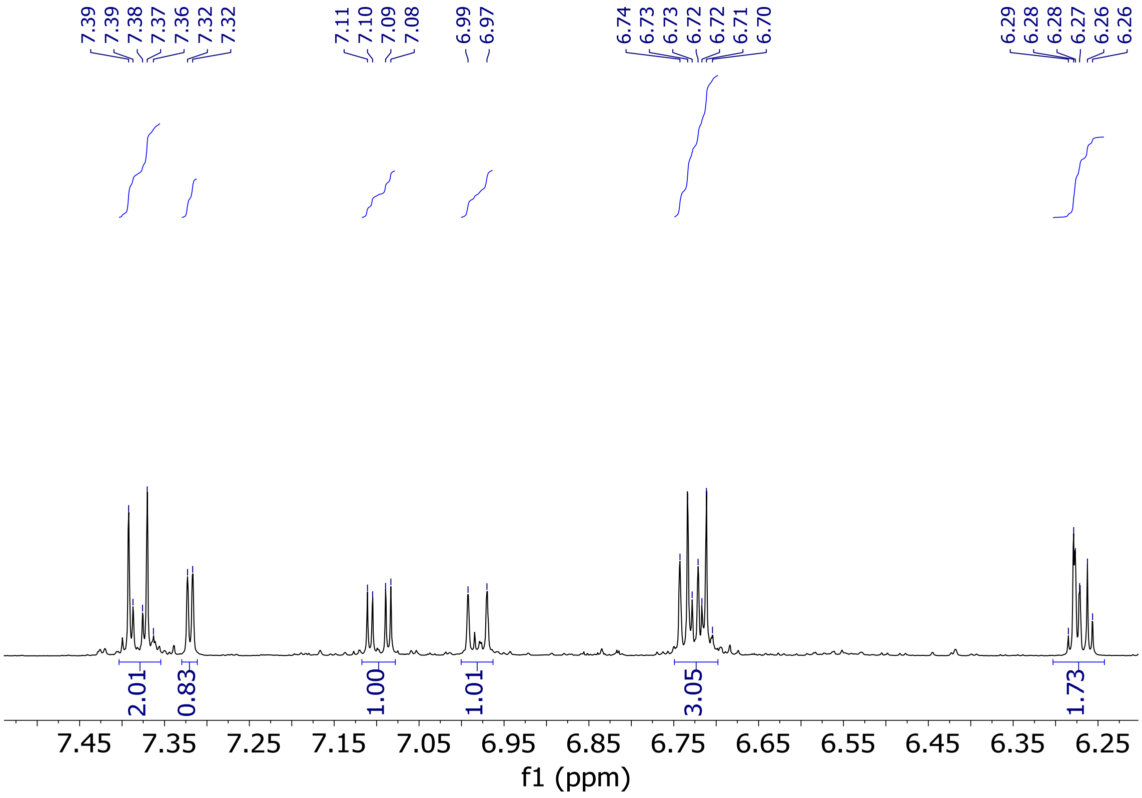

Figure S5a. ^{1}H-NMR spectrum of analog **8** in CD_3OD. (7.5–6.2 ppm ampliation).

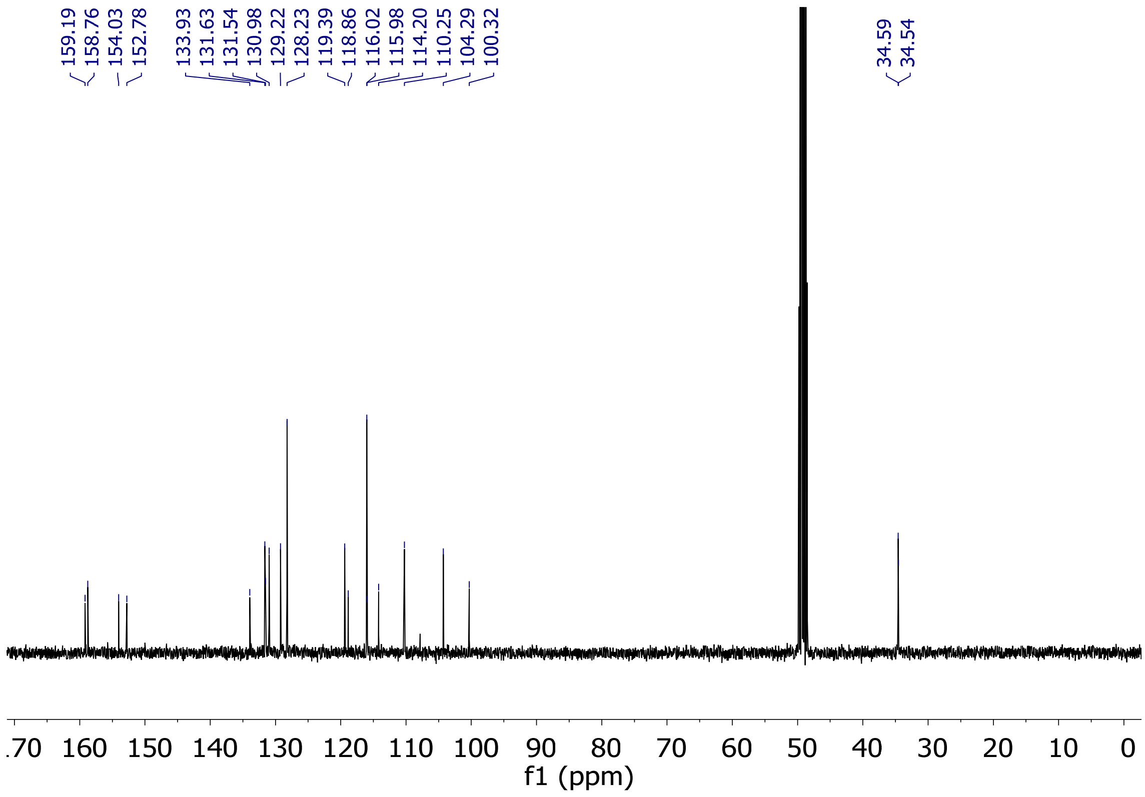

Figure S6. ^{13}C-NMR spectrum of analog **8** in CD_3OD.

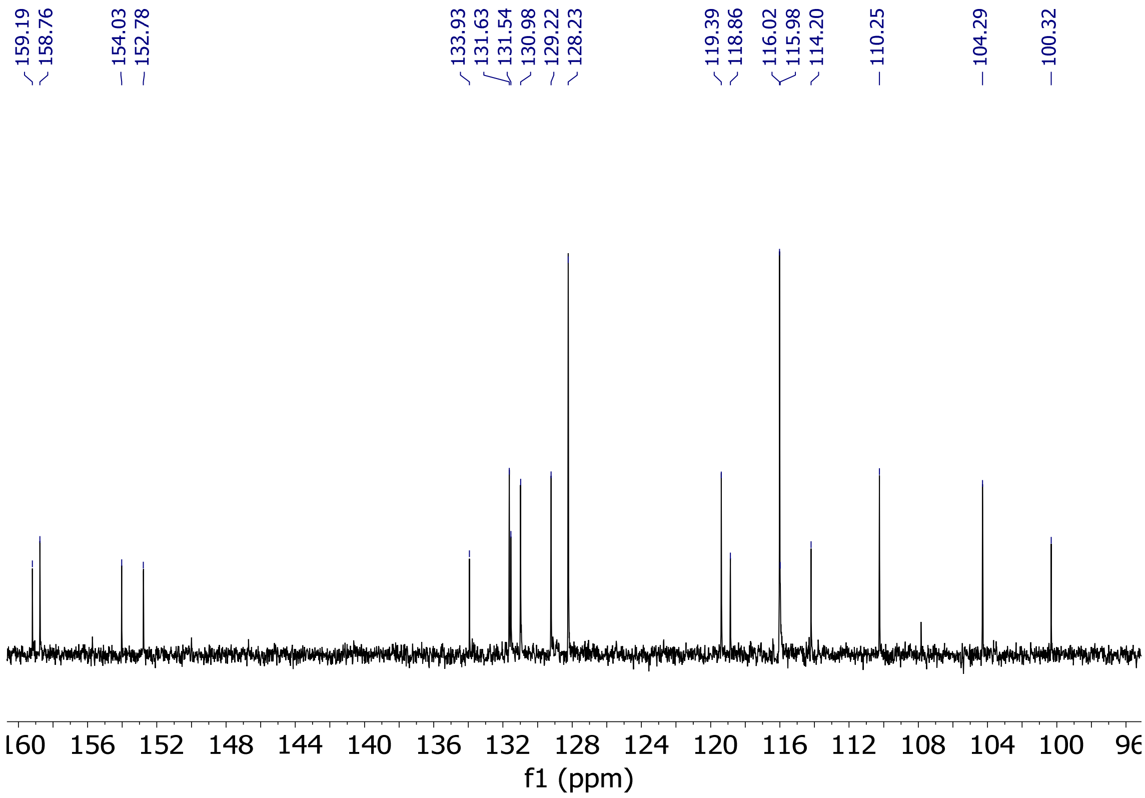

Figure S6a. ^{13}C-NMR spectrum of analog **8** in CD_3OD (160–96 ppm ampliation).

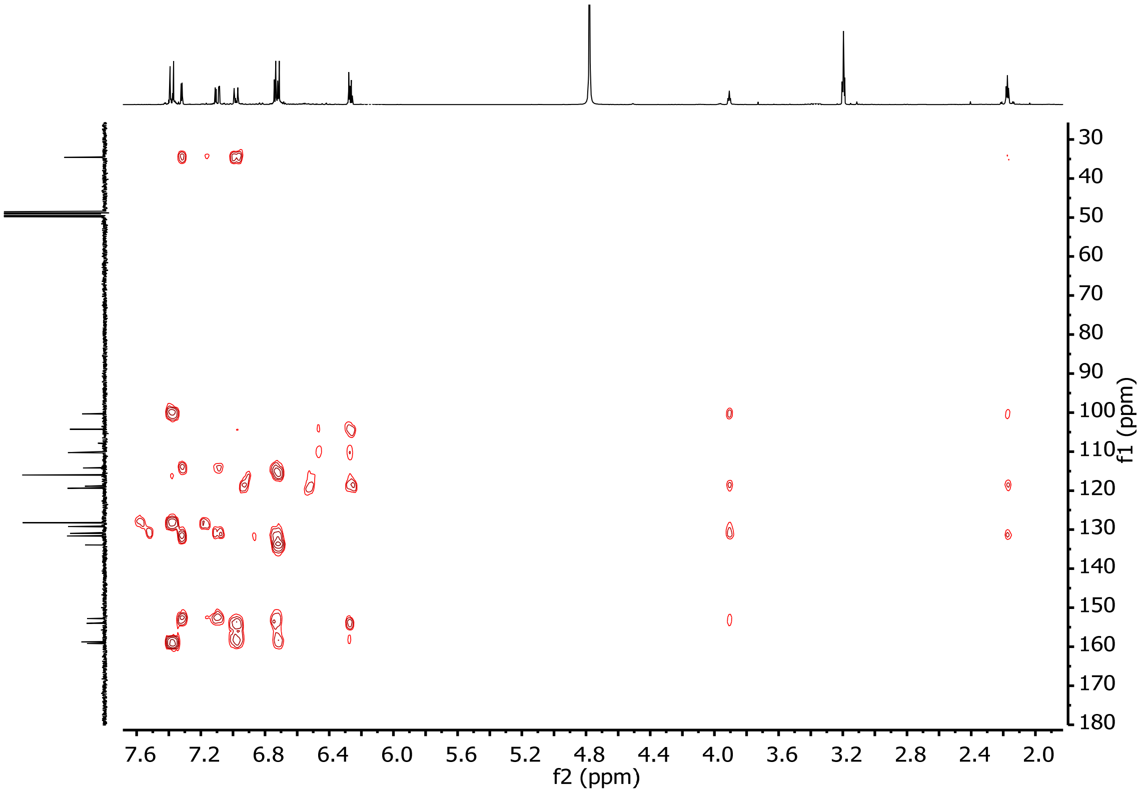

Figure S7. ^{1}H-^{13}C-HMBC spectrum of analog **8** in CD_3OD.

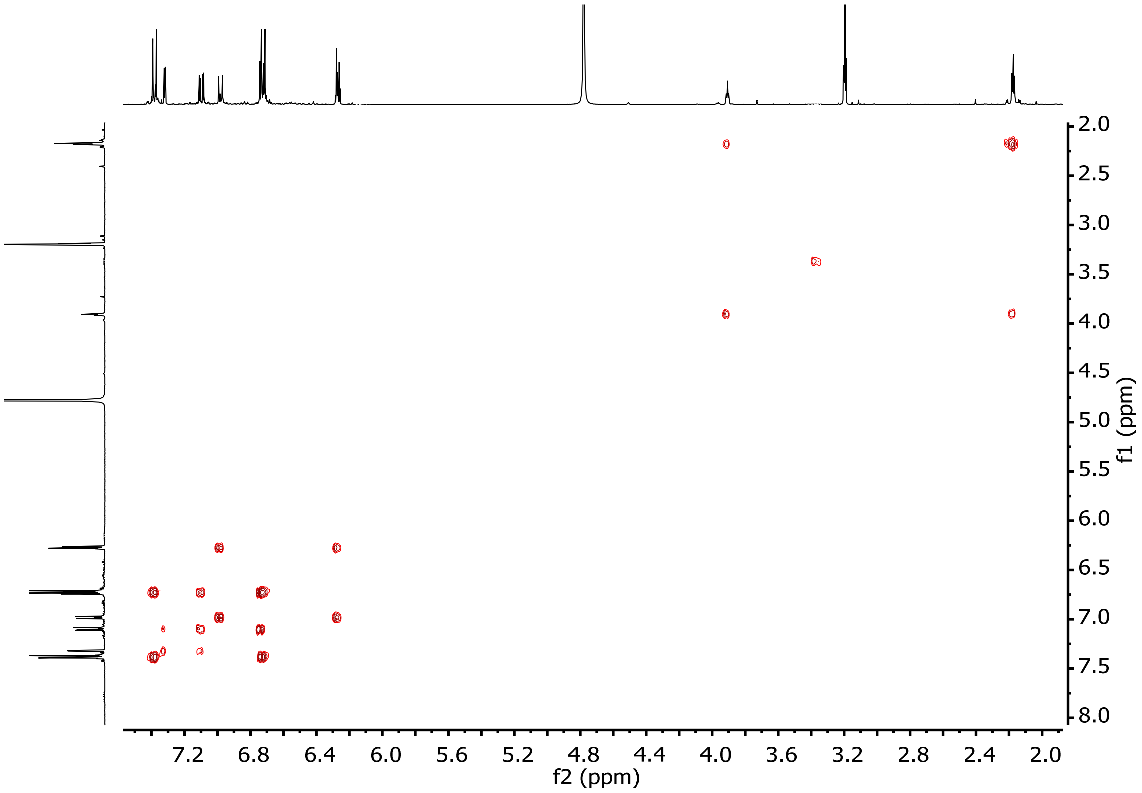

Figure S8. ^{1}H-^{1}H-COSY spectrum of analog **8** in CD_3OD.

Table S8. ^{1}H-NMR and ^{13}C-NMR full peak assignment of analog **8** in CD_3OD.

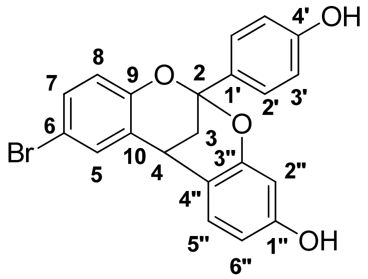

**Position**[a]	**^{1}H /ppm (J/Hz)** MeOD	**^{13}C /ppm** MeOD	**HMBC** MeOD
1	-	-	-
2	-	100.3	2, 2′, 3, 4
3	2.10-2.24 (*m*)	34.5	2, 10, 4′′
4	3.91(*t*, *J* = 3.3 Hz)	34.6	5, 9, 3′′, 5′′
5	7.32 (*d*, *J* = 2.5 Hz)	131.0	4, 7, 9
6	-	114.2	8
7	7.10 (*dd*, *J* = 8.6, 2.5 Hz)	131.6	5, 9
8	6.70-6.75 (*ov*)	119.4	6, 10
9	-	152.8	4, 5, 7
10	-	131.5	3, 8
1′	-	133.9	3′
2′	7.35-7.40 (*m*)	128.2	2, 4′
3′	6.70-6.75 (ov)-	116.0	1′
4′	-	159.2	2′
1′′	-	158.8	5′′
2′′	6.30-6.24 (*ov*)	110.2*	4′′, 6′′
3′′	-	154.0	4, 5′′
4′′	-	118.9	3, 6′′
5′′	6.98 (*d*, *J* = 8.9 Hz)	129.2	1′′, 3′′, 4
6′′	6.30-6.24 (*ov*)	104.3*	2′′, 4′′

[a]Numbering was done according to the flavylium structure. * these signals could be interchangeable.
